# Rita Oladele: shining a light on invasive fungal pathogens

**DOI:** 10.2471/BLT.23.030323

**Published:** 2023-03-01

## Abstract

Rita Oladele talks to Gary Humphreys about the need for more investment in invasive fungal pathogen surveillance, research and clinical capacity.

*Q: You are best known for your work on invasive fungal pathogens. Could you start by explaining what is meant by that term?*

A: It’s used to describe infections other than those affecting the skin or mucosal tissue. Invasive fungal infections include infections of the bloodstream which can impact the immune system or otherwise cause serious systemic harm. 

*Q: You trained as a clinician in Lagos. How did your interest in invasive fungal infections develop?*

 A: After medical school I went to the University College Hospital, Ibadan, to complete my training, and a childhood friend diagnosed with acute lymphocytic leukemia was brought into the unit where I was working as a medical officer. Part of my job was administering intravenous drugs and so I had the responsibility of giving my friend the chemotherapy drugs she needed. She developed a fever, and on the presumption that she had picked up a bacterial infection, we started her on antibiotics. But the fever would not come down and despite changing the antibiotics again and again the fever persisted. I noticed a rash in my friend’s mouth which I identified as oral thrush, which is caused by the fungus *Candida albicans,* and we administered statin drops. The rash cleared up, but then it came back and so did the fever and despite all our efforts to bring it down, my friend died. It was a shock to me and for a long time, as a young doctor, I wondered if it was the *Candida* that had killed her by getting into her bloodstream. That wasn’t something that had been investigated at that time, and at Manchester University, I devoted my doctoral thesis to the question. I was able to grow *Candida* in blood culture samples from 12 of the 230 critically ill patients in a trial I set up. Eleven of those 12 patients died. That was the beginning of my journey in the world of invasive fungal pathogen research.

*Q: There is increasing concern regarding the spread of invasive fungal infections. Is that concern justified?*

A: To the extent that increased attention drives research into invasive fungi and the diseases with which they are associated it is certainly helpful, but it is important to note that the epidemiological picture is by no means clear, largely because of inadequate reporting and surveillance. It is sometimes reported that invasive fungal infections are responsible for around 1.5 million deaths worldwide each year, but the truth is we don’t really know what the disease burden is. A review of studies reporting fungal infection rates from Nigeria that I did with Professor David Denning in 2014 indicated that an estimated 11.8% (20.5 million of 174.0 million) of the Nigerian population suffer from a serious fungal infection each year. However, as we noted at the time of publication, epidemiological studies are required to validate or modify these estimates. I personally have been working towards that end since then, notably in antigen screening studies that I have done on the prevalence of infections with *Cryptococcus neoformans* (a common yeast) in people living with HIV.

“[Cryptococcal meningitis] is assessed to be the second leading cause of death among people living with HIV.”

*Q: Why focus on that particular pathogen and sub-population?*

A: Because of the association between the two. *Cryptococcus* is found in soils worldwide, and for most people, exposure to it is harmless, but for people with compromised immune systems such as those with HIV it can cause serious and sometimes life-threatening disease. Symptoms are usually mild at first, so infection often goes unnoticed or is misdiagnosed, but as the pathogen spreads from the lungs to the brain the disease can become severe. Cryptococcal meningitis, which can result in excruciating headaches, blindness, deafness and often death, is estimated to account for up to 15% of HIV-related deaths globally and as much as 50% in resource-limited settings. It is assessed to be the second leading cause of death among people with HIV.

*Q: Is the association between invasive fungal infections and immune deficiency common?*

A: Yes, in fact, there is a strong association between most invasive pathogens and immune deficiency, another good example being the association between *Aspergillus* infection and tuberculosis. *Aspergillus* is a common mould, and most people breathe in its spores every day without getting sick, but post-therapy tuberculosis patients can develop chronic pulmonary aspergillosis, and in Nigeria we often see tuberculosis/*Aspergillus *co-infections. Nigeria ranks among the countries with the highest burden of tuberculosis (accounting for 4.4% of the estimated 10.6 million people falling ill with TB in 2021), so this is of particular concern for us. There also appears to be an association between invasive fungal pathogens and diseases or conditions that can impact immune responses, including diabetes, malnutrition and certain genetic disorders. 

*Q: Which fungal pathogens are of greatest concern in Nigeria?*

A: *Cryptococcus* *neoformans* and *Aspergillus* are of concern for the reasons given, but we tend to see more invasive *Candida* infections than anything else. People tend to associate *Candida* with relatively harmless yeast infections, but in fact there are no fewer than five *Candida* species on the WHO Fungal Priority Pathogens List (WHO FPPL) including two – *Candida auris *and* Candida albicans* – that are numbered among the fungi considered to be of critical concern – the highest concern category. *Candida auris* is a relative newcomer, first emerging in the Republic of Korea in the late 1990s and then in Japan in 2009. Since then, it has shown up in countries around the world, and appears to spread very easily in health-care settings. One of the main concerns with *Candida auris* is the fact that it is showing resistance to the most commonly used antifungal medications (azoles, echinocandins, polyenes and pyrimidines). WHO has started monitoring the incidence of *Candida* bloodstream infections in 23 countries, and GLASS (WHO's Global Antimicrobial Resistance and Use Surveillance System) will soon be collecting resistance data on isolates from *Candida* bloodstream infections.

*Q: Have you seen any cases of Candida auris infection in Nigeria?*

A: A handful of *Candida auris* infections were identified in blood culture samples taken from four patients in four different hospitals here in 2022. Three of the patients were in the intensive care unit and one was critically ill in a gynaecology ward. We followed up on those findings with a study to look at the potential reservoirs of the organism in the patients’ environment. We didn’t find any *Candida auris,* but we did find pathogenic fungi in places that would normally be considered ‘clean’ if not sterile. This is a finding that was recently confirmed by a postgraduate student who I tasked with establishing the prevalence of fungal pathogens in the water being used in hospitals. The student sampled watersheds from which the hospitals drew their water to see if there was contamination with fungal pathogens, and found fungi everywhere.

“[We found] pathogenic fungi in places that would normally be considered ‘clean’ if not sterile.”

*Q: One of the aims of WHO in publishing the WHO FPPL was to increase attention to fungal pathogens. Why have they been neglected in the past?*

A: Partly because no one looks for them. Clinicians in our hospitals do not routinely suspect or look for fungal infections when they are developing a diagnosis. That being the case, they do not request that laboratories isolate them in the samples they send. There is also a shortage of laboratories able to test for the presence of fungal pathogens in samples. In Nigeria, there are just a handful of laboratories, including our centre here in Lagos. 

*Q: What can be done to address these two challenges?*

A: First, we need to train clinicians to be on the alert for cases of invasive fungal infection, to initiate treatment as early as possible and make sure cases get reported. This can be achieved with the right investment, as borne out by the work we have done in collaboration with the CDC Foundation (a United States of America-based independent nonprofit that mobilizes philanthropic and private-sector resources to support the Centers for Disease Control and Prevention’s prevention work). The CDC Foundation made a commitment along with seven other public health organizations working in Africa to end deaths from cryptococcal meningitis in Africa by 2030. 

After we completed the cryptococcosis study, we worked with the support of the CDC Foundation to train more than 700 health-care workers who are now able to make effective rapid diagnoses and manage patients. This not only benefited patients, it also helped with monitoring. The clinicians we trained reported 250 cases of cryptococcal meningitis to the national data repository in the six months following the initiative. Before that, no cases had ever been recorded. 

It is important to note that the research we carried out also drove innovation in cryptococcal meningitis prevention. We showed that by screening for *Cryptococcus* antigens you can identify at-risk patients and initiate pre-emptive antifungal therapy. We were also able to demonstrate that it was cost-effective to screen advanced HIV patients and used this data to drive our advocacy. Now almost every facility that manages advanced HIV patients routinely screens for cryptococcosis. 

*Q: Where you will be focusing your efforts in the coming years?*

A: I will continue to push for greater awareness and investment, notably through the Medical Mycology Society of Nigeria*, *a multidisciplinary society that I helped found. The society brings together a range of researchers, clinicians, students, and laboratory scientists from across a range of specialties including neurosurgery, and can play a key role in directing efforts towards research and programmes most likely to benefit the at-risk population in Nigeria. On the advocacy side, I will be working to advance a range of agendas, including ensuring access to effective diagnostics and therapies for invasive fungal infections. These need to be included in our essential diagnostics and medicines lists and as part of our universal health coverage basic package. 

